# Prediction of mental effort derived from an automated vocal biomarker using machine learning in a large-scale remote sample

**DOI:** 10.3389/frai.2023.1171652

**Published:** 2023-08-03

**Authors:** Nick Taptiklis, Merina Su, Jennifer H. Barnett, Caroline Skirrow, Jasmin Kroll, Francesca Cormack

**Affiliations:** ^1^Cambridge Cognition, Tunbridge Court, Cambridge, United Kingdom; ^2^Department of Psychiatry, Herschel Smith Building for Brain & Mind Sciences, University of Cambridge, Cambridge, United Kingdom; ^3^Department of Psychological Science, University of Bristol, Bristol, United Kingdom

**Keywords:** computerized cognitive assessment, voice markers, automated speech recognition, remote testing, voice-based assessment, cognitive load, mental effort

## Abstract

**Introduction:**

Biomarkers of mental effort may help to identify subtle cognitive impairments in the absence of task performance deficits. Here, we aim to detect mental effort on a verbal task, using automated voice analysis and machine learning.

**Methods:**

Audio data from the digit span backwards task were recorded and scored with automated speech recognition using the online platform NeuroVocalix^TM^, yielding usable data from 2,764 healthy adults (1,022 male, 1,742 female; mean age 31.4 years). Acoustic features were aggregated across each trial and normalized within each subject. Cognitive load was dichotomized for each trial by categorizing trials at >0.6 of each participants' maximum span as “high load.” Data were divided into training (60%), test (20%), and validate (20%) datasets, each containing different participants. Training and test data were used in model building and hyper-parameter tuning. Five classification models (Logistic Regression, Naive Bayes, Support Vector Machine, Random Forest, and Gradient Boosting) were trained to predict cognitive load (“high” vs. “low”) based on acoustic features. Analyses were limited to correct responses. The model was evaluated using the validation dataset, across all span lengths and within the subset of trials with a four-digit span. Classifier discriminant power was examined with Receiver Operating Curve (ROC) analysis.

**Results:**

Participants reached a mean span of 6.34 out of 8 items (SD = 1.38). The Gradient Boosting classifier provided the best performing model on test data (AUC = 0.98) and showed excellent discriminant power for cognitive load on the validation dataset, across all span lengths (AUC = 0.99), and for four-digit only utterances (AUC = 0.95).

**Discussion:**

A sensitive biomarker of mental effort can be derived from vocal acoustic features in remotely administered verbal cognitive tests. The use-case of this biomarker for improving sensitivity of cognitive tests to subtle pathology now needs to be examined.

## Introduction

Computerized test batteries have improved reliability of cognitive testing by eliminating sources of human error through standardized and automated administration and scoring (Zinn et al., 2021). Recent developments in accuracy of automatic speech recognition (ASR) software mean that voice-based computerized assessments are now practicable and scalable (Taptiklis et al., 2017). Speech is the response modality for a broad range of widely used traditional neuropsychological tests tapping into a range of cognition functions. Using speech as a vocal biomarker is particularly appealing because it can be easily obtained using smartphones, thus increasing accessibility whilst requiring minimal resources and costs compared to in-clinic assessments (Quatieri et al., 2015).

Speech planning and execution is a complex and uniquely human behavior including cognitive and motor components, involving input from language, speech, and motor areas of the brain, and careful orchestration of vocal and respiratory motor functions. This yields a rich canvas of vocal features, which include paralinguistic features (e.g., pauses, breathing, stuttering), prosodic features (e.g., pitch, rhythm, intensity, and rate of speech), and voice quality features (e.g., irregularities in pitch or intensity, croakiness, breathiness). Vocal features change under increased task demand, for example when participants are doing two tasks at the same time, they show more variable or shorter silence periods, and increased pitch and volume (Segbroeck et al., 2014; Lopes et al., 2018). Speech data has emerged as a non-invasive measure of cognitive load and may prove a valuable extension to tests in which vocal responses are already required (Mijić et al., 2017).

Cognitive load refers to the mental demand a particular task imposes on the human cognitive system for a specific person (Paas and Van Merriënboer, 1994). This can be separated into mental load (properties of the task difficulty or demand and environment), mental effort (capacity or resources allocated to the task), and task performance (resulting from the interaction between mental load and mental effort; Paas and Van Merriënboer, 1994). Two people can obtain the same test results with different levels of mental effort (Paas et al., 2003); one person may need to work laboriously, whereas for another minimal effort may be required. Moreover, having prior knowledge or skills related to the task may result in a decrease on cognitive demand (Borghini et al., 2017). Thus, performance metrics may only provide a crude estimate of cognitive function since they are likely to decline only when task demands exceed capacity. An accurate measure of mental effort can furnish important additional information that is not reflected in simple performance metrics (Paas et al., 2003).

A measure of mental effort may be particularly helpful for increasing sensitivity to neurodegenerative disorders, where patients may perform in the normal range on cognitive tests in the presence of brain degeneration or pathology in the earlier stages of disease progression (Gregory et al., 2017). As pathology progresses and available cognitive resources decrease, greater mental effort is required to maintain a given level of performance. Increases in mental effort may eventually become insufficient to maintain performance, leading to a decrease in task performance (Ranchet et al., 2017). Augmentation of mental effort is therefore likely to precede and predict measurable incident cognitive decline on neuropsychological testing (Aurtenetxe et al., 2013; Ahmadlou et al., 2014). Metrics of mental effort may therefore help to increase the sensitivity of cognitive testing to more subtle decline or impairment.

Indices of cognitive load have more recently been captured with physiological measurements, including heart rate, skin conductance, pupil dilation, eye blinks, and movement and EEG (Lopes et al., 2018). Physiological measures consistently show increased cognitive load in healthy older adults compared with younger adults when performing the same task, and similarly increased cognitive load for patients with Mild Cognitive Impairment (suggested as an intermediate state between normal aging and dementia (Petersen and Morris, 2005) compared to healthy aging (Ranchet et al., 2017). Using such measures may provide a specific indication of mental effort with sensitivity and precision in hypothesis testing and validity (van Gog et al., 2010; Ayres et al., 2021). EEG studies have been commonly used to predict mental effort based on network connectivity and spectral features (Friedman et al., 2019). Imaging and physiological methods have clear advantages compared to less sensitive subjective methods (Sweller et al., 2011). However, since these measures require specialized equipment, scalability is limited. Self-report scales, which enquire about perceived mental effort and task difficulty, are more readily scalable, however these correlate poorly with one another and with task performance metrics (DeLeeuw and Mayer, 2008). Another promising avenue for measuring mental effort is the use of vocal features captured during task performance. Instantaneous data can be recorded in a non-intrusive setting using simple devices such as smartphones. Data from small samples under experimental settings manipulating cognitive load, have shown the ability to distinguish mental effort based on various voice parameters, and analyzed using machine learning classifiers (Yin et al., 2008; Segbroeck et al., 2014; Magnúsdóttir et al., 2017; Mijić et al., 2017). Nonetheless, further work is needed to validate vocal features as a measure of cognitive load and this necessitates larger datasets to improve detection accuracy. Detection accuracy is particularly important for application in clinical populations and in early detection of cognitive impairment where precision is often required on an individual level. Moreover, exploring the feasibility of conducting an experimental voice study in a remote setting is warranted as this enables the cost-effective inclusion of a larger sample with less participant burden using common technology such as personal computers and smartphones. As such, the current study describes the development of a fully automated and device-agnostic verbal cognitive assessment system capable of remote web-based assessment, with which we aim to classify mental effort during a task of increasing difficulty using automated voice analysis and machine learning. We report data from a large sample of participants tested in their own homes, with which we aim to develop, test and then validate a novel voice biomarker of mental effort and cognitive load.

## Methods

### Participants

Participants were recruited via the crowdsourcing platform Prolific (Palan and Schitter, 2018) between November 2018 and January 2019. Participants met the following eligibility criteria: aged between 17 and 90 years, English speaker, no history of language problems and never diagnosed with mild cognitive impairment or dementia. All subjects were reimbursed £2.10 for their time. All subjects provided consent for data collection and were informed of their rights to cease participation or withdraw at any time.

### Procedure

Prospective participants were directed to the study homepage, which provided an explanation of the study and gave the opportunity to consent or decline participation. Data was collected on participants' own devices. They were instructed to turn on the sound and enable audio recording. Participants were instructed to perform the tasks on their own, in a quiet room and to the best of their ability; and not to complete these tests if unusually stressed, tired or unwell, or under the influence of alcohol or other substances. Instructions and questions were presented, and responses were required in the English language.

Participants provided basic demographic information, including age, sex, native language, country of residence, country of origin, and educational level [categorized as follows: completed formal education at (1) Middle/Junior High, (2) High School, (3) Higher Education (4) Postgraduate Education]. They also responded to self-report questionnaires of mood and pain.

A verbal cognitive test battery was administered, modeled on traditional neuropsychological assessments and adapted for automated administration. Tests were administered via the assessment platform NeuroVocalix^TM^ in the described order: digit span forwards, digit span backwards, verbal paired associates and serial subtraction, taking just under 20 min on average. Task instructions were delivered through verbal prompts from the speakers, accompanied by matching visual prompts displayed on the screen. During tasks, verbal stimuli were delivered auditorily only via device speakers. Raw audio data were recorded from participants' own devices as 16-bit mono Pulse Core Modulation (PCM) at a sample rate of 16 KHz. Information on devices, browsers and operating systems used during testing were collected automatically via the User-Agent header of HTTP requests sent by the web-browsers on participants' devices.

### Cognitive assessment

The current study focuses on digit span backwards, a test in which the number of items to be held in the active memory buffer is incrementally increased, thereby increasing cognitive load. A sequence of numbers is presented (e.g., “2-7-3-9”), which participants are asked to repeat in reverse (“9-3-7-2”). The task begins with the presentation of only two digits. When a participant successfully completes a trial of a given length, they then move onto the next trial which presents a sequence with one additional digit, up to a maximum sequence length of 8. The task terminates early when participants fail on three consecutive attempts of the same sequence length. The sequence of numbers for each digit span trial was fully randomized to avoid providing the opportunity for machine learning algorithms to learn to differentiate digit sequences and not cognitive load. The importance of this randomization is discussed in detail in Mijić et al. (2017).

Responses were scored online during task administration. This was evaluated via an Automatic Speech Recognition (ASR) proxy system developed in-house, which accessed multiple systems simultaneously (including IBM Watson, Amazon Lex, and Google Cloud speech-to-text systems) and fine-tuned these technologies to improved accuracy, reliability, and speed of response detection. This enabled automated scoring and implementation of task continuation/discontinuation rules during administration. Voice data was recorded and stored for analysis and quality control. For each trial, the recording window remained open until any of these conditions were reached: (1) a correct response was detected by any ASR; (2) at least two ASRs agreed on an incorrect response of the expected span (equivalent to the number of digits presented); (3) contiguous silence of length 2^*^span+2 s had been reached; or (4) an absolute window duration of 2.5^*^span+2 s had been reached.

### Statistical analysis

Participants' voice responses were recorded and analyzed on a trial-by-trial basis. The NeuroVocalix platform records each trial as a separate audio file. Audio features from each trial were extracted using the openSMILE Version 2.1.0 feature extraction toolkit (Eyben et al., 2013). This toolkit extracts a wide array of vocal features suitable for signal processing and machine learning analyses (Mijić et al., 2017). The toolkit was configured to use 10 ms moving window, a time period where vocal features can be considered stationary (Rao, 2011), and the “emo_large” feature set was selected. This feature set contains features which are derived from spectral and prosodic characteristics, mel-frequency cepstral coefficients and harmonic which are then aggregated across each audio sample by applying a series of summary statistics (e.g., means, variance, distances). The combination of these features improves the robustness of the system (China Bhanja et al., 2019). A single acoustic feature vector of all 6,552 features in this feature set was derived for each trial audio recording.

Analysis was completed using Anaconda Python version 3.7. The acoustic feature vectors were normalized within each participant, with resulting variables expressing within-subject trial-by-trial deviations from the within-subject average across trials. This means that within-subject differences in voice in relation to differences in task difficulty could be examined. Participants were excluded if they were unable to reach a span of three. Maximum backwards digit span therefore ranged from 3 to 8 items, and only correct responses were included in onward analyses. A personalized measure of cognitive load was calculated for each trial by dividing the trial digit span by the maximum span attempted and dichotomized with “high load” defined as trials that were >0.6 of each participant's maximum span attempted, and “low load” as trials below this threshold.

Data were divided into training (60% of sample), test (20%), and validate (20%) datasets, with different participants in each set. Training and validation data were used in model building and evaluating model accuracy, respectively. Data modeling was completed with five different machine learning classifiers using scikit-learn 0.23.1 (Pedregosa et al., 2012). Due to the large sample size in this study, it was possible to explore multiple alternative classifiers. Models were trained to predict the binary cognitive load categorization based on acoustic features alone. The models utilized in the current study were:

- Logistic regression (LR): is a linear model for classification, with the probabilities describing outcomes modeled using a sigmoid or logistic function. The logistic regression model was implemented with a “sag” optimization algorithm suitable for large datasets and maximum number of iterations for solvers to converge of up to 1,000.
- Naïve Bayes (NB): is a supervised learning method based on Bayes' theorem, which assumes feature independence, that is that the presence of a feature in a class is unrelated to other features. The likelihood of features is assumed to be Gaussian, this classifier can deal with modeling outliers very well but a limitation of this approach is that it performs well for small vocabulary (Tóth et al., 2005; Bhangale and Mohanaprasad, 2021) which is sufficient for the current study.
- Support vector machine (SVM) with linear kernel is an algorithm which finds linear combinations that best separate outcomes It has been widely applied to classifying voice data with high accuracy rates (Aida-zade et al., 2016) and capacity to deal with higher dimensionality (Sonkamble and Doye, 2008). The algorithm was specified with a regularization parameter of C = 15.0, a primal optimization problem, a loss function specified as the square of the hinge loss, and an “l1” penalty leading to sparse coefficient vectors. Tolerance for stopping criteria was specified as 0.01. For all other parameters the default settings were selected.
- Random forest (RF) classifier: This is an ensemble machine learning algorithm, representing a combination of decision trees. This method was chosen as it is relatively robust to non-linear data, noise, and can support high-dimensional data with redundant features (Boateng et al., 2020). This meta estimator fits several decision tree classifiers on the dataset and uses averaging to improve the predictive accuracy and control over-fitting. Bootstrap samples of the dataset were used to build each tree with the quality of the split measured through gini importance criteria. A minimum sample required to split an internal node was specified at 10, and split points at any depth were only considered if they left a minimum training sample of eight in both left and right branches. The maximum number of features for each split was set at 0.1. One hundred trees were built in the forest.
- Gradient boosting (GB): is an estimator which utilizes integer-based data structures (histograms) instead of relying on sorted continuous values when building the trees. GB has been found to be superior to other proposed machine learning models but involves more computation and training time (Dash et al., 2022). The size of the trees was controlled by specifying minimum 10 samples per leaf, a minimum of 6 samples per split, a maximum tree depth of 4. This was completed with a combination of gradient boosting with bootstrap averaging (bagging). At each iteration the base classifier was trained on a fraction of 0.75 subsample of the available training data, which is drawn without replacement with the size of features in the subset specified as a maximum of 0.65. The number and contribution of weak learners was controlled by the parameters n_estimators specified at 200 and learning_rate specified at 0.1.

Different models can confer different sensitivities (Mijić et al., 2017). The goal of the analyses of test data was to identify the model that best generalizes to new data (e.g., generalize from the training and validation dataset to predict an independent test dataset; Yarkoni and Westfall, 2017). Performance of the different machine learning classifiers were examined in test data, and model classifier discriminant power was estimated using Receiver Operating Characteristic (ROC) curve analysis. The Area Under the ROC Curve (AUC) is a combined measure sensitivity and specificity, which provides a summary measure of accuracy, which supports the interpretation of the goodness of a classification algorithm evaluated, whilst not being influenced by the selection of specific decision thresholds or cut-offs (Hajian-Tilaki, 2013). Since the AUC provides the average value of sensitivity for all the possible values of specificity (Hajian-Tilaki, 2013), it allows for the direct comparison of classifier discriminant power of different algorithms.

The validation dataset was held out for final model evaluation using the most predictive algorithm. As cognitive load increases with span length, so do the duration of utterances for each trial. It was necessary to exclude the possibility that classification accuracy was not merely dependent on utterance length. In the validation test sample we therefore explored accuracy of our most predictive model within all data and again after limiting responses to a span length of four, median span score, where trials were approximately equally likely to be categorized as high and low load.

Minimum sample size for the validation test dataset was calculated on the basis of test performance at a digit length of four, using methods previously described (Buderer, 1996). A 5% level of significance (two-sided), and a width of the 95% confidence interval at 10% were specified, and prevalence of high cognitive load in this data was specified at 50%. Percentage accuracy of cognitive load classification from a range of vocal characteristics has been found to be around 70% (Yin et al., 2008). With estimates of 70% sensitivity and 70% specificity, a sample of 370 participants were required for the validation dataset.

## Results

### Participants

Testing and audio data were acquired from 3,074 participants (mean age: 34.2, range 17–86; 1,161 male, 1,914 female). ASR did not identify any correct responses for 307 participants and two additional participants did not reach a backward span of at least three items. Manual quality checks on these data from participants indicated that these had excessive background noise (talking or television in the background) or other poor audio quality. These data were excluded, leaving an analysis set of 2,764 participants. Included participants differed modestly from those that were excluded in terms of age, with excluded participants being slightly older (mean age 35.8 vs. 34.1, *t* = −2.33, *p* = 0.02), and with men proportionally more likely to be excluded (12% men vs. 8% women, χ^2^ = 7.25, *p* = 0.007), but with no differences between included and excluded participants in relation to education (χ^2^ = 4.64, *p* = 0.20).

Participants included in the onward analysis were aged between 17 and 36, with a mean of 34.1 years of age (SD = 12.3). The majority of participants (76.4%, *n* = 2,111) were resident in countries with English as a main language, primarily the UK and USA and spoke English was their first language (75.5%, *n* = 2,088). One hundred and twenty-one individuals reporting a language other than English as their first language, and data on first language were unavailable for 555 participants. Demographic information is provided in Table 1.

**Table 1:** Participant socio-demographic details.

		**Number of participants**	**Percentage (%)**
Sex	Male	1,022	37.0
	Female	1,742	63.0
Education level	Middle/Junior High	35	1.3
	High School	557	20.2
	Higher Education	1,537	55.6
	Postgraduate	635	23.0
First language	English	2,088	75.54
	Other	121	4.38
	Missing	555	20.08
Occupational status	Full-time employed	1,029	37.23
	Part-time employed	503	18.20
	Student, not in employment	270	9.77
	Unemployed and job seeking	104	3.76
	Not in paid work (homemaker, retired or disabled)	216	7.815
	Other	56	2.03
	Missing	586	21.20

### Devices and operating systems

Participants were successfully tested on a range of platforms; nearly half (46%) completed the testing on a Microsoft Windows platform, followed by mobile phone (iPhone and Android; 36%), then by Apple Mac (13%).

### Digit span performance

**Figure 1:**
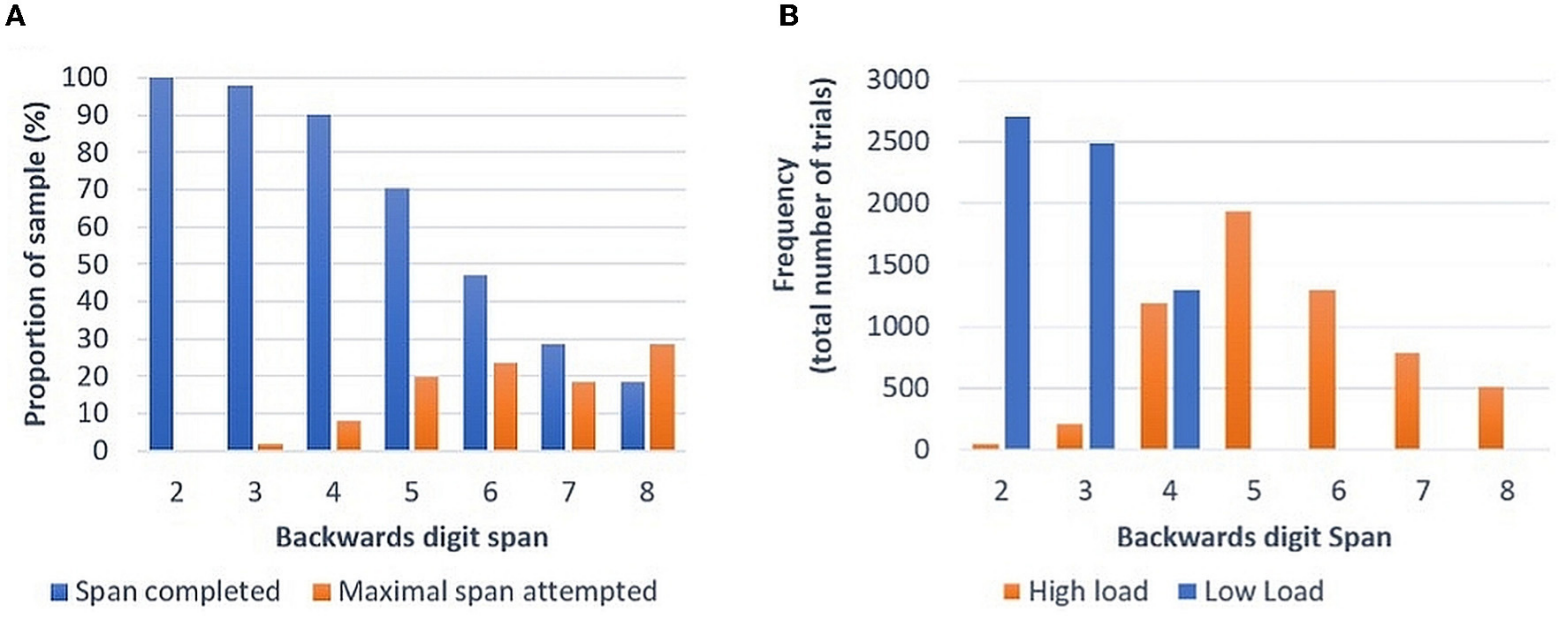
**(A)** Proportion of sample successfully completing each span length and maximal span attempted. **(B)** Frequency of allocation of different span utterances to high and low load.

As expected, performance declined as number of items to be recalled increased (Figure 1A). Overall participants reached a mean digit span backwards of 6.34 out of a total of eight items (SD = 1.38). Calculating “high load” at >0.6 of participants' maximum span attempted, a roughly even split between high (*n* = 231) and low load (*n* = 276) trials included in analyses was seen at a digit span of 4 (Figure 1B). The maximal digit span attempted was not correlated with age (Spearman's Rho: 0.02, *p* = 0.26), with a broadly equivalent degree of trial complexity attempted across the age-range of the sample.

### Machine learning

#### Sample allocation

Participants allocated into train, test, and validate samples were similar with regards to distribution of sexes, education, and mean age. A similar proportion of low and high load voice responses were assigned as train, test, and validate datasets. Table 2 provides details on voice responses and participant characteristics for these datasets.

**Table 2:** Disposition of participants and voice data across training, test and validate datasets.

**Dataset**	**Number of participants (%)**					**Mean age (SD)**	**Number of voice responses (%)**		
	**Total**	**Sex**		**Education completed at**			**Total**	**Low load**	**High load**
		**Female**	**Male**	** ≤ Age 18**	**>Age 18**				
Train	1,658	1,064 (64.17)	594 (35.83)	356 (21.47)	1,302 (78.52)	33.99 (12.05)	7,414	3,862 (52.09)	3,552 (47.91)
Test	553	321 (58.05)	232 (41.95)	117 (21.16)	436 (78.84)	33.90 (12.77)	2,532	1,306 (51.58)	1,226 (48.42)
Validate	553	357 (35.56)	196 (35.44)	119 (21.52)	434 (78.48)	34.45 (12.29)	2,555	1,325 (51.86)	1,230 (48.14)

#### Model test data

**Figure 2:**
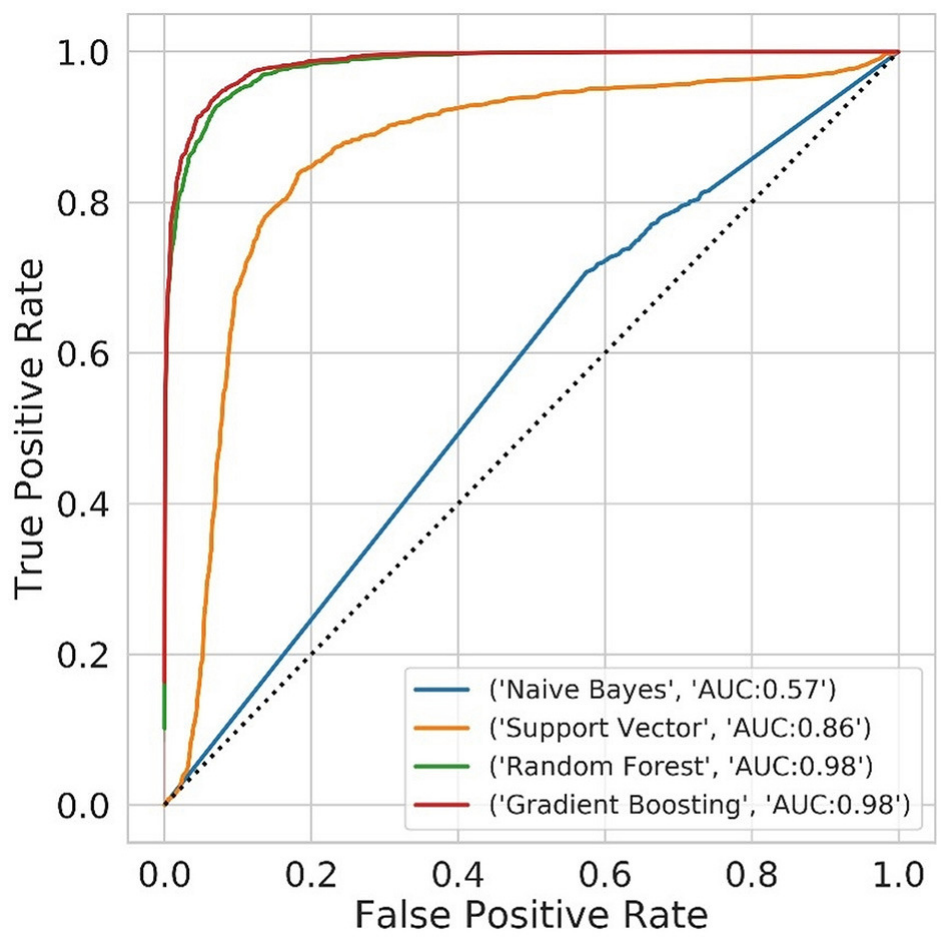
Random Forest and Gradient Boosting had the highest accuracy and area under the curve (AUC) on the test dataset.

Results of the different classification models on test data are shown in Table 3. The logistic regression model did not converge and is therefore not reported. Receiver Operating Curves are presented in Figure 2. The best performing models were Random Forest and Gradient Boosting, with a modestly higher accuracy for Gradient Boosting. These are both ensemble models which are robust to high-dimensional datasets with correlated and redundant features.

**Table 3:** Performance of all machine learning classifiers in predicting cognitive load for test and validate samples, and Gradient Boosting classifier for the full validation sample, and after limiting to a span length of four.

**Data**		**Classification**	**Performance**				
**Sample allocation**	**Spans included**	**Machine learning classifier**	**Precision**	**Recall**	**f1-score**	**Accuracy**	**AUC**
Test	All	Naïve Bayes	0.57	0.56	0.55	0.56	0.57
	All	Support vector	0.82	0.82	0.82	0.82	0.86
	All	Random forest	0.93	0.93	0.93	0.93	0.98
	All	Gradient boosting	0.94	0.94	0.94	0.94	0.98
Validation	All	Gradient boosting	0.94	0.94	0.94	0.94	0.99
	4-digit only	Gradient boosting	0.87	0.86	0.86	0.86	0.95

Correct responses only were included in analyses.

#### Model validation

Results of the Gradient Boosting Classifier on the held-out data showed that accuracy remained high, with an AUC of 0.99 for the full validation dataset, and an AUC of 0.95 for the validation data when limited to spans at a length of four digits. Results of the classification models on validation datasets are shown in Table 3. This shows that the most predictive features in this classifier comprised Mel-Frequency Cepstrum Coefficients and spectral features.

**Figure 3:**
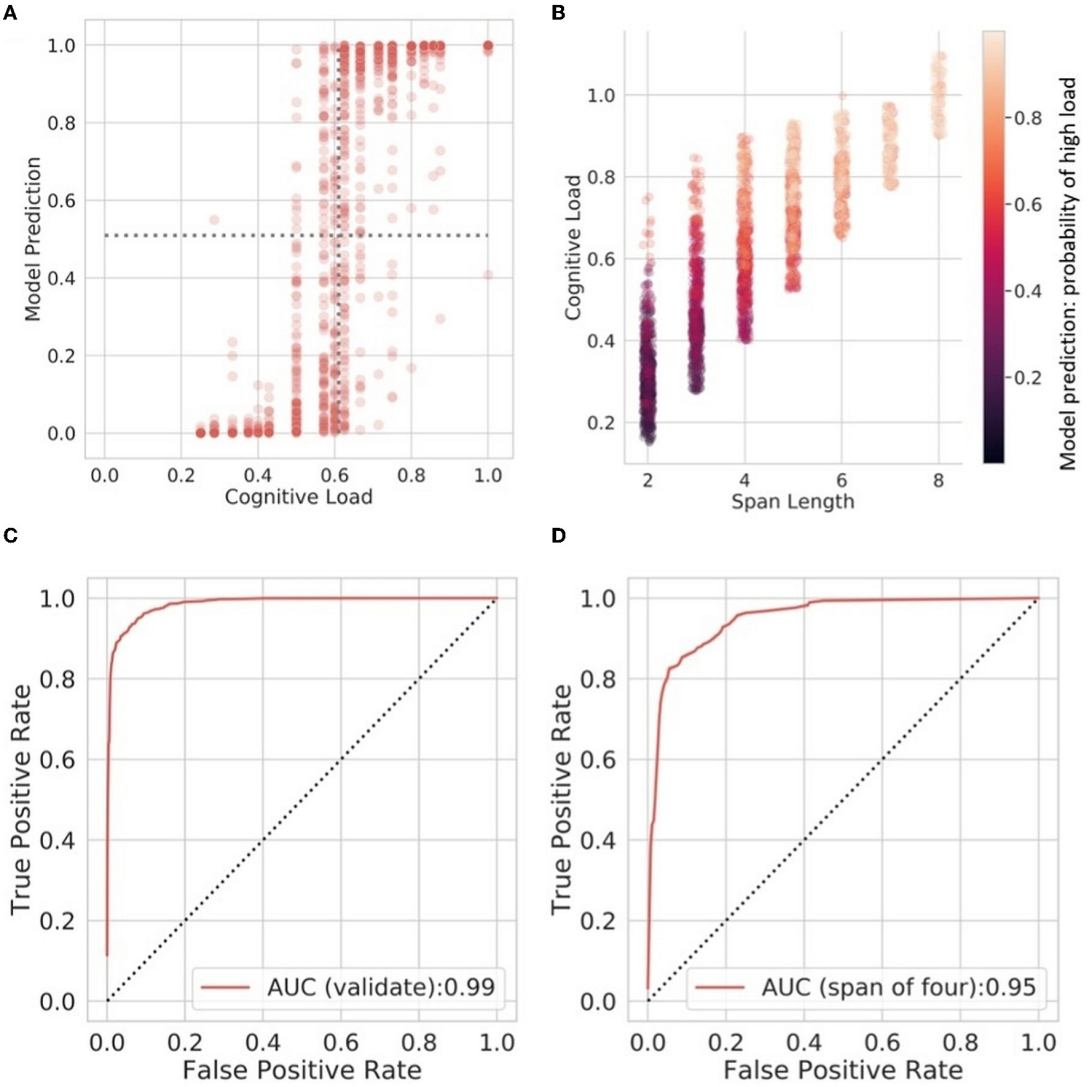
**(A)** The relationship between model probability prediction of cognitive load and the observed load in the validation data. The boundary between high and low cognitive load is 0.6. The decision boundary for the model is 0.5. **(B)** Scatterplot showing the relationship between load, span length, and model probability prediction. **(C, D)** ROC curves for the Gradient Boosting classifier in the full validation data set, and at a span of four digits, respectively.

Classification accuracy is shown in Figure 3A, which shows the relationship between model probability prediction of cognitive load and the observed load in the validation data. Figure 3B shows how these probability predictions relate to span length and cognitive load. This shows that even shorter utterances (e.g., digit spans of 2 or 3) are accurately identified as high load when these near the top-end of performance levels for individual participants. Receiver Operating Curves for the validation data are shown for the full data (Figure 3C) and in data limited to a span length of four (Figure 3D).

## Discussion

The current study validated a machine learning classifier which reliably identifies high and low mental effort from acoustic voice data obtained during neuropsychological testing, in trials where test performance is otherwise undifferentiated. These results are in line with other voice papers measuring cognitive load, demonstrating the feasibility of using vocalics as a biomarker for mental effort (Yin et al., 2008; Meier et al., 2016; Mijić et al., 2017). Accuracy rates of ~68% were reported by Mijić et al. (2017) on an arithmetic task using several machine learning methodologies such as support vector machine and neural networks. Similar results were found in Stroop and reading measures of speech-based cognitive load with accuracy as high as 77.5% using a Gaussian Mixture Model (GMM) classifier (Yin et al., 2008). The high accuracy rates achieved in the current study may be due to the use of an adaptive task, which enabled us to personalize utterances as high or low load during training allowing us to increase sensitivity and accuracy. Prior work trained models using static labels of task difficulty that are dependent on the task performed rather than an individuals' ability to perform the task. Using this biomarker provides information regarding the interaction between task difficulty and participant characteristics and can provide more nuance in cognitive data collected than the blunt pass/fail diagnostics commonly in use. This highly accurate cognitive load differentiator was derived from data collected remotely on participants' own devices and in their own homes, requiring neither specialist equipment nor study supervision, showing excellent scalability.

The findings suggest that the characterization of mental effort in our healthy adult sample was not related to co-occurring vocal and cognitive changes related to aging, since performance on the task was not associated with age in our sample. The validation of our findings within a sub-sample of data with a backwards span of only four, shows that the algorithm maintains high accuracy when controlling for utterance length. Looking beyond basic performance data and obtaining insight into mental effort may be particularly helpful in research aiming to identify more subtle impairments or progressive decline. This may be particularly applicable to research examining cognitive deterioration and dementia. In Alzheimer's disease, pathophysiological changes begin years, if not decades before diagnosis of clinical dementia (Sperling et al., 2011). Evidence suggests that abnormal biomarkers, commonly obtained using neuroimaging techniques or medical procedures, such as amyloid beta (Aß) positivity, can precede measurable cognitive impairment or decline using standard cognitive tests (Elman et al., 2020). As research moves to intervene in presymptomatic phases of the disease, measures that are sensitive to early disease-related changes are required (Donohue et al., 2014).

Lack of correspondence between brain pathology and clinical manifestations of brain damage is commonly attributed to related constructs of cognitive reserve and scaffolding, thought to attenuate cognitive decline and mask disease severity (Valenzuela and Sachdev, 2006; Gregory et al., 2017). These models describe active compensation in the brain either through recruiting new and alternate brain networks less susceptible to disruption or by enlisting compensatory approaches (Stern, 2006; Park and Reuter-Lorenz, 2009). It is theorized that as disease burden increases, progressive pathology eventually overwhelms compensatory functions and performance starts to deteriorate. This means that overt cognitive deficits are likely to occur after significant changes to the efficacy with which cognitive task performance is achieved. Neurophysiological studies support the notion that compensatory mechanisms are enlisted in fulfilling cognitive tasks in patients with Alzheimer's Disease and Mild Cognitive Impairment (Ranchet et al., 2017).

Furthermore, common challenges in the voice-based literature include speaker variability, such as pronunciation, sex and speech rate, external noise, and channel variability where different smartphones and microphones are used (Forsberg, 2003). Such confounds are particularly impactful in smaller datasets often used in the cognitive load literature. As such, one of the strengths of this study is the large sample size employed which is made possible by the fully automated scoring system using four ASR engines. To the best of our knowledge, this is the first study measuring mental effort using ASR that was completely remote using a novel web-based application. The ability to capture such a large dataset allowed us to use different individuals in the training and test datasets, thus training the classifiers on a wide range of voices and incorporating sources of channel variability and noise thus increasing the generalizability of the model. The majority of studies in this area utilize much smaller samples, and often different cross-validation schema subsequently not being able to ensure that part of the test dataset has not also been used for training (Trabelsi et al., 2022). Lastly, the current study used an adaptive task, the backwards digit span, which allowed us to personalize cognitive load to determine optimal individual differentiation between low and high workload.

Our results suggest that automatically administered and scored verbal cognitive tests can be used to concurrently generate both reliable measures of performance and useful vocal biomarkers of mental effort. Changes in vocal features have been revealed as potentially sensitive markers for a range of clinical conditions, including frontotemporal dementia (Nevler et al., 2017) and Parkinson's disease (Benba et al., 2016). Overall research has indicated that vocal characteristics can provide valuable insights into mental effort. In line with previous research, the current study demonstrates that vocal biomarkers can assist in accurately identifying trials characterized by high cognitive load and generalize to novel data where task performance is intact, but mental effort is high. Further work is now required to replicate our findings within clinical populations, to examine the sensitivity of vocal digital biomarkers of mental load to the presence and progression of neurodegenerative pathology.

## Code availability statement

Analysis code is available from the authors on request.

## Data availability statement

The raw data supporting the conclusions of this article will be made available by the authors, without undue reservation.

## Ethics statement

Ethical review and approval was not required for the study on human participants in accordance with the local legislation and institutional requirements. The patients/participants provided their written informed consent to participate in this study.

## Author contributions

NT, FC, JB, and MS conceived of and designed the study. NT, MS, and FC conducted the data collection. FC, NT, MS, JK, and CS conducted data analysis. All authors contributed to writing and review of the manuscript. All authors contributed to the article and approved the submitted version.
